# Distribution and role of high‐risk human papillomavirus genotypes in women with cervical intraepithelial neoplasia: A retrospective analysis from Wenzhou, southeast China

**DOI:** 10.1002/cam4.1559

**Published:** 2018-05-30

**Authors:** Yuli Wang, Jisen Xue, Xinyue Dai, Lulu Chen, Junli Li, Yancheng Wu, Yan Hu

**Affiliations:** ^1^ Department of Gynecology The 1st Affiliated Hospital Wenzhou Medical University Wenzhou China

**Keywords:** association, cervical intraepithelial neoplasia, genotype distribution, human papillomavirus

## Abstract

To add the growing literature on baseline of high‐risk human papillomavirus (HR‐HPV) genotype distribution in cervical intraepithelial neoplasia (CIN) before the widespread using of HPV vaccines in Chinese mainland and to improve risk stratification of HR‐HPV–positive women. Retrospectively, the data of age, cervical HPV genotypes, cytology, and pathology were collected from 1166 patients who received loop electrosurgical excision procedure (LEEP). HPV genotypes were analyzed with Flowcytometry Fluorescence Hybridization Method. And then HPV prevalence, HR‐HPV genotype distribution and the correlation of HR‐HPV genotypes with CIN2+ (CIN2 or severer) were analyzed. The role of multiple HR‐HPV types infection with or without HPV16/18 in the pathogenesis of CIN2+ was also analyzed. The 6 most common HR‐HPV genotypes were HPV16, 58, 52, 33, 18, and 31 in descending order. Compared to HR‐HPV–negative women, HPV16, 33 or 58 positive women had higher risk of CIN2+ (OR = 5.10, 95% CI = 2.68‐9.70; OR = 3.09, 95% CI = 1.39‐6.84; OR = 3.57, 95% CI = 1.85‐6.89, respectively). And women who were infected by multiple HR‐HPV types infection with HPV16/18 also had higher risk of CIN2+ (OR = 2.58, 95% CI = 1.35‐4.92). However, multiple HR‐HPV types infection without HPV16/18 did not increase the risk significantly (*P* = .08). Compare to bivalent Cervarix^®^ and quadrivalent Gardasil^®^, HPV prophylactic vaccine targeting HPV31, 33, 52, and 58 might provide women more protection from HPV‐induced cervical cancer in China. The women who infected by HPV16, 33, 58, or multiple HR‐HPV types with HPV16/18 have higher risk of CIN2+ and need to be paid more attention in screening processes. And the role of multiple HR‐HPV types infection without HPV16/18 needs be further identified in more studies.

## 1 INTRODUCTION

Cervical cancer is the fourth most common cancer and the fourth leading cause of death among females globally.^1^ As a country with a large population, data of China from GLOBOCAN reported that there were 61 691 new cases and 29 526 deaths because of cervical cancer in 2012.^1^ And persistent high‐risk human papillomavirus (HR‐HPV) infection is the most important cause in the progress of premalignant and malignant epithelial lesions of cervix.^2^ HPV genotypes including HPV16, 18, 31, 33, 35, 39, 45, 51, 52, 56, 58, 59, 68, 73, and 82 are defined as HR‐HPV.^3^ And it has been suggested that the potential carcinogenicity of these genotypes is different.^4^ It showed that HPV16 and 18 were responsible for approximately 55% to 60% and 10% to 15% of cervical cancer, respectively.^5^ Other than HPV16 and 18, some studies confirmed HPV31, 33, 35, 45, 52, and 58 as the most frequently detected genotypes in invasive cervical cancer (ICC).^6^, ^7^ Therefore, new HPV genotyping tests are emerging to help improve risk stratification of HR‐HPV‐positive women in cervical screening programs.^8^

As for primary prevention of cervical cancer, HPV prophylactic vaccine has been licensed by Food and Drug Administration (FDA) since 2006.^9^ Many studies have proven that bivalent Cervarix^®^ and quadrivalent Gardasil^®^ are highly safe, well tolerated, and available with almost no severe side effects when given to adolescent and preadolescent females prior to first sexual intercourse.^10^ The 2 vaccines potentially offer highly efficacious protection against ICC caused by HPV16 and/or 18 (HPV16/18).^10^ Since 2014, 9‐valent HPV (9vHPV) vaccine has been approved to provide protection against HPV6, 11, 16, 18, 31, 33, 45, 52, and 58.^11^ A retrospective cross‐sectional study reported that the inclusion of 5 additional HR‐HPV genotypes (HPV31, 33, 45, 52, and 58) would increase the protection to almost 90% of the infection responsible for cervical cancer.^12^ Except that, the efficacy and immunogenicity of 9vHPV vaccine were confirmed by a randomized, international, double‐blind study.^13^ Based on the efficiency and safety of HPV vaccines, bivalent Cervarix^®^ has been introduced in Chinese mainland in 2017 to reduce the burden of HPV‐related disease.^14^ Before that, standard data on the genotype distribution of HR‐HPV infection in cervical intraepithelial neoplasia (CIN) are important to estimate the likely effectiveness of bivalent prophylactic vaccination for cervical cancer prevention and to provide an outlook on the development of second‐generation HPV vaccines.

As to secondary prevention of cervical cancer, HR‐HPV test has been used as preliminary screening with cytology for 14 years.^5^ Cytology united with HR‐HPV test, colposcopy, and biopsy could be summarized as three‐step strategy according to the American Society for Colposcopy and Cervical Pathology (ASCCP) guideline.^5^ Based on the pathological results of colposcopy‐directed biopsy (CDB), patients are selected to be treated. For the women diagnosed with CIN2 or severer (CIN2+) by CDB, a diagnostic excisional procedure such as loop electrosurgical excision procedure (LEEP) is recommended generally.^5^ LEEP has been proven to be feasible, tolerable and have favorable postoperative outcomes accompanied by providing satisfied samples.^15^, ^16^ By studying these screening results, many investigators showed the prevalence and genotype distribution of HPV,^17^, ^18^ the association of HPV with women's sociodemographic status,^19^ abnormal cytology,^20^ or histology.^21^ However, the relative contribution of HPV16/18 in infection with multiple HPV types to the occurrence of CIN2+ is little studied, which just become the main difference of the present study from others.

This study aimed at providing a robust estimate of HPV prevalence and HR‐HPV genotype distribution in CIN before the widespread using of HPV vaccines in Chinese mainland by analyzing the screening outcomes of 1166 patients who received LEEP retrospectively. In addition, to improve risk stratification of HR‐HPV–positive women, the role of specific HR‐HPV genotypes, and multiple HR‐HPV types infection with or without HPV16/18 in the progress of CIN would be shown.

## 2 MATERIALS AND METHODS

### 2.1 Population

Retrospectively, this study included 1166 women who underwent LEEP and were diagnosed with CIN subsequently at the 1st Affiliated Hospital of Wenzhou Medical University between January 2012 and December 2015. These women were the ones who had complete results of cytology, HPV typing, and pathology of cervix including biopsy and LEEP, had no use of vaginal medication or no sexual intercourse in the previous 3 days before sample collection, had not been vaccinated against HPV infection before, had not underwent any medical or invasive treatment for CIN previously, and were not immunosuppressive or immunodeficient. And to ensure the results from same test methods, the part of women using other test methods was excluded. Finally, age, results of screening tests, and final pathologic outcomes of CDB and LEEP were documented from patients' files. This study was approved by the Hospital Ethics Committee of the 1st Affiliated Hospital of Wenzhou Medical University. As a retrospective study, written consents were not required.

### 2.2 Cytology

Cervical samples were collected using a disposable cervical brush (Ningbo HLS Medical Products Co., Ltd.) after menstruation, and then the samples were stored and transported in PreservCyt solution for thinprep cytologic test (TCT) using ThinPrep 2000 system (Hologic Inc.). The results were classified into 7 groups: negative for intraepithelial lesion or malignancy (NILM), atypical squamous cells of undetermined significance (ASC‐US), low‐grade squamous intraepithelial lesions (LSIL), atypical squamous cells‐cannot exclude high‐grade squamous intraepithelial lesions (ASC‐H), high‐grade squamous intraepithelial lesions (HSIL), atypical glandular cells (AGC), and carcinoma (including squamous cell carcinoma and adenocarcinoma) based on the Bethesda System of 2001,^22^ while unsatisfactory results were excluded.

### 2.3 HPV genotyping test

HPV genotypes were analyzed with Flowcytometry Fluorescence Hybridization Method (Tellgen, Shanghai, China) using Nucleic acid genotyping kit for human papillomavirus according to the manufacturer's instructions. A disposable sterile cervical sampler (Jiangsu Jianyou medical technology Co., Ltd.) was used to collect cervical exfoliated cell. The samples were analyzed within 1 week from collection. Using a suspension bead array method, HPV genotypes were differentiated. The experimental procedure had been described in previous study including DNA extraction, PCR amplification, and hybridization.^23^ This method could differentiate 26 HPV genotypes including 18 HR‐HPV genotypes (HPV16, 18, 26, 31, 33, 35, 39, 45, 51, 52, 53, 56, 58, 59, 66, 68, 73, and 82) and 8 low risk HPV (LR‐HPV) genotypes (HPV6, 11, 40, 42, 44, 55, 61, and 83). In our study, regardless of LR‐HPV, the cases with HR‐HPV infection were divided into different groups or subgroups according to different characteristics.

### 2.4 Histology

The referral indications for CDB were mainly based on ASCCP guideline.^5^ In all 1166 women, only 65 women cotesting no HPV16/18 infection, cytology NILM, and 43 women cotesting no HR‐HPV infection, cytology ASC‐US were referred to CDB because of patients' wish for diagnosis of cervical lesion after the persistent infection of HR‐HPV for more than 2 years or the clinical manifestation, such as recurrence of contact bleeding. Exposuring the utmost transformation zone, CDB was carried out using 4% acetic acid and iodine solution to show suspected cervical lesions where biopsies were performed subsequently. And random multiple biopsies were taken if there was no specific focus under the visual field. Endocervical curettage was used in the patients who were infected by HPV18, whose cytologic result was AGC or whose colposcopic examination was unsatisfied.

The referral indications for LEEP were primarily based on the results of CDB. In addition, women were selective to referral for LEEP in following cases: unsatisfactory colposcopic vision combined with suspicion of HSIL, patients' wish for maximum safety after the persistent infection of HR‐HPV for more than 2 years, reduplicated of abnormal cytology and the clinical manifestation, such as recurrence of contact bleeding. And it was performed using a diathermal electrocauterizer with a wire loop which size was determined by colposcopic examination of lesions. The specimen was fixed in formalin and then sent to pathology department.

According to the World Health Organization criteria,^24^ the grades of CIN (CIN1‐3) were diagnosed. The highest grade histologic result obtained from either CDB or LEEP was defined as the final histologic grade of cervix. In present study, CIN2+ included CIN2 and CIN3. CIN1 was used as control. Unknown any screening status, the pathologists provided the initial diagnosis of biopsies and surgical specimens. And the final pathological result was reviewed by a chief pathologist. Any contentious results would be unified by discussion.

### 2.5 Statistical analysis

Statistical analyses were performed with SPSS software version 19.0 for Windows. Results were presented as means ± standard deviations (*SD*), numbers, or frequencies as appropriate. Chi‐square test was used to evaluate the association between HR‐HPV infection and CIN2+. Student's *t* test was used to evaluate the association between age and CIN2+. Kruskal‐Wallis test was used to evaluate the association between cytology and CIN2+. Univariate analysis and multivariate logistic regression analysis were used to find the association between HR‐HPV and CIN2+. Odds ratios (ORs) with 95% confidence intervals (CIs) were calculated when risks were estimated. *P* value corresponded to two‐sided tests and *P* < .05 was considered as statistically significant.

## 3 RESULTS

Categorical data were summarized descriptively in Table 1. The mean age of 1166 patients was 42.5 ± 9.8 years old (range 21‐82). In all 1166 women, the cytological results of NILM, ASC‐US, LSIL, ASC‐H, HSIL, and AGC accounted for 11.7% (136/1166), 38.9% (454/1166), 27.4% (319/1166), 5.7% (67/1166), 15.9% (185/1166), and 0.4% (5/1166) respectively, and there was no carcinoma. The overall prevalence of detectable HPV was 94.9% (1107/1166). The prevalence of HR‐HPV was 93.8% (1094/1166) which was obviously higher than LR‐HPV (84/1166, 7.2%). Moreover, the prevalence of infection with single and multiple HR‐HPV type was 70.1% (817/1166) and 23.8% (277/1166) respectively. The most prevalent genotype of HR‐HPV was HPV16 (421/1166, 36.1%). HPV58, 52, 33, 18, and 31 were next only to HPV16 accounted for 23.4% (273/1166), 17.2% (200/1166), 11.7% (137/1166), 6.4% (75/1166), and 5.6% (65/1166), respectively. It was worth noticed that the infection cases of previous 6 HR‐HPV genotypes were amounted for 85.3% (995/1166). Setting histology as the gold standard, there were 247 (21.2%) CIN1 and 919 (78.8%) CIN2+ consisted of 561 CIN2, 358 CIN3.

**Table 1:** Clinical characteristics of patients( n = 1166)

Categories	Subcategories	Value/n	Overall prevalence, %
Age, mean ± SD (range)		42.5 ± 9.8 (21‐82)	
Cytology	NILM	136	11.7
	ASC‐US	454	38.9
	LSIL	319	27.4
	ASC‐H	67	5.7
	HSIL	185	15.9
	AGC	5	0.4
HPV testing	HPV negative	59	5.1
	HPV positive	1107	94.9
	LR‐HPV infection	84	7.2
	HR‐HPV infection	1094	93.8
	Single HR‐HPV infection	817	70.1
	Multiple HR‐HPV infection	277	23.8
Top 6 HPV genotypes	HPV16	421	36.1
	HPV58	273	23.4
	HPV52	200	17.2
	HPV33	137	11.7
	HPV18	75	6.4
	HPV31	65	5.6
Histology^a^	CIN1	247	21.2
	CIN2	561	48.1
	CIN3	358	30.7

AGC, atypical glandular cells; ASC‐H, atypical squamous cells‐cannot exclude high‐grade squamous intraepithelial lesions; ASC‐US, atypical squamous cells of undetermined significance; CIN1, cervical intraepithelial neoplasia 1; CIN2, cervical intraepithelial neoplasia 2; CIN3, cervical intraepithelial neoplasia 3; HPV, human papillomavirus; HR‐HPV, high‐risk HPV; HSIL, high‐grade squamous intraepithelial lesions; LSIL, low‐grade squamous intraepithelial lesions; LR‐HPV, low risk human papillomavirus; NILM, negative for intraepithelial lesion or malignancy.

a The highest grade histologic result between colposcopy‐directed biopsy and loop electrosurgical excision procedure was chosen as the final histologic result.

### 3.1 Distribution of HR‐HPV genotypes by cytologic status

The number and percent of women infected by the top 6 HR‐HPV genotypes in all grades of abnormal cytology were shown in Table 2. The most common genotype of HR‐HPV was HPV16. HPV58 was the second most common type in abnormal cytology. HPV52 was more common in NILM (29/136) than other abnormal cytology.

**Table 2:** HPV genotypes in cases of CIN by cytologic diagnosis

HPV genotypes	Cytologic diagnosis (n = 995)					
	NILM (n = 136)	ASC‐US (n = 454)	LSIL (n = 319)	ASC‐H (n = 67)	HSIL (n = 185)	AGC (n = 5)
HPV16	59 (43.4)	137 (30.2)	103 (32.3)	31 (46.3)	90 (48.6)	1 (20.0)
HPV18	14 (10.3)	36 (7.9)	16 (5.0)	4 (6.0)	5 (2.7)	0 (0.0)
HPV31	9 (6.6)	29 (6.4)	11 (3.4)	4 (6.0)	11 (5.9)	1 (20.0)
HPV33	9 (6.6)	46 (10.1)	47 (14.7)	5 (7.5)	28 (15.1)	2 (40.0)
HPV52	29 (21.3)	92 (20.3)	48 (15.0)	8 (11.9)	23 (12.4)	0 (0.0)
HPV58	18 (13.2)	116 (25.6)	81 (25.4)	16 (23.9)	41 (22.2)	1 (20.0)

AGC, atypical glandular cells; ASC‐H, atypical squamous cells‐cannot exclude high‐grade squamous intraepithelial lesions; ASC‐US, atypical squamous cells of undetermined significance; HPV, human papillomavirus; HSIL, high‐grade squamous intraepithelial lesions; LSIL, low‐grade squamous intraepithelial lesions; NILM, negative for intraepithelial lesion or malignancy.

Data are number (%).

### 3.2 Distribution of HR‐HPV genotypes by histologic status

**Figure 1:**
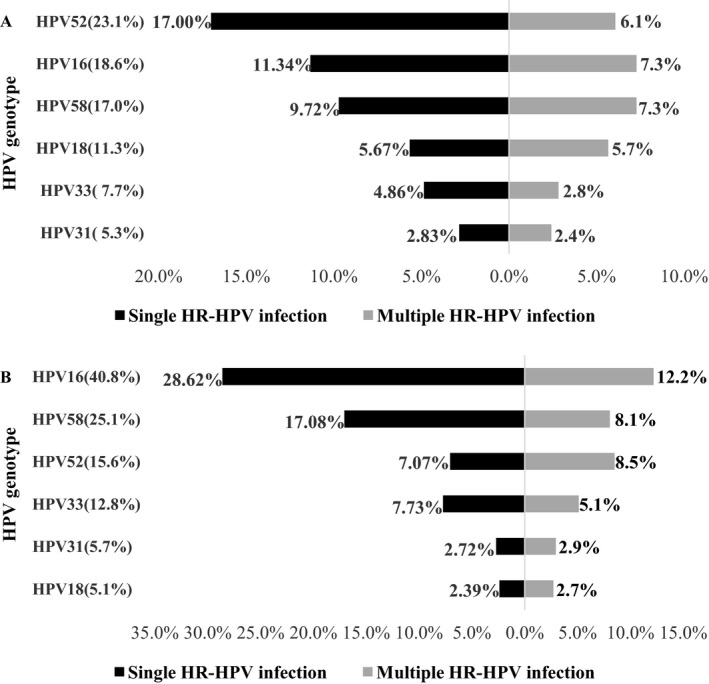
Percent of women infected by the top 6 HR‐HPV genotypes in CIN1 (n = 247) and CIN2+ (n = 919) cases. CIN 1 cases were shown in A, and CIN 2+ cases were showed in B. HR‐HPV, high‐risk human papillomavirus; CIN, cervical intraepithelial neoplasia; CIN2+, CIN2 or severer

The percent of women infected by the top 6 HR‐HPV genotypes in CIN1 and CIN2+ cases was shown in Figure 1. In 247 CIN1 cases, the most common genotype of HR‐HPV was HPV52 (57/247, 23.1%). The next 5 common genotypes in decreasing order were HPV16 (46/247, 18.6%), 58 (42/247, 17.0%), 18 (28/247, 11.3%), 33 (19/247, 7.7%), and 31 (13/247, 5.3%). However, the most common genotype of HR‐HPV found in 919 cases with CIN2+ was HPV16 (375/919, 40.8%). HPV58, 52, 33, 31, and 18 were accounted for 25.1% (231/919), 15.6% (143/919), 12.8% (118/919), 5.7% (52/919), and 5.1% (47/919), respectively. Their combined contribution was 88.9% (817/919) in CIN2+.

### 3.3 The association between HR‐HPV and CIN2+

The women with CIN1 had the lowest positive rate for HR‐HPV as 89.5% (221/247), followed by CIN2 (529/561, 94.3%), and the women with CIN3 had the highest positive rate as 96.1% (344/358). There was significant difference in these 3 groups (χ^2^ = 11.45, *P* = .00).

To minimize the bias caused by small sample size when comparing risk factor profiles, we excluded the 5 cases with cytology‐AGC from correlation analysis. Table 3 showed that no significant difference was found in the prevalence of CIN2+ by age (*P* = .45). And there was significant difference found in groups with different cytologic results (*P* = .00). To investigate whether different HR‐HPV genotype and infection with multiple HR‐HPV types were risk factors for CIN2+, the patients were classified to 3 types of grouping. In type 1, there were HR‐HPV–positive (72/1161) and HR‐HPV–negative group (1089/1161). In type 2, there were 7 groups: HR‐HPV–negative (72/797) and single type infection with HPV16 (290/797), 18 (36/797), 31 (31/797), 33 (81/797), 52 (107/797), or 58 (180/797). In type 3, there were 4 groups: HR‐HPV–negative (72/675), single type infection with HPV16 or 18 (326/675), coinfection with HPV16/18 (156/675), and coinfection without HPV16/18 (121/675). And significant differences were found among the groups in these 3 types of grouping mentioned above (*P* = .00).

**Table 3:** Univariate analysis of risk factors for CIN2+

Categories	Subcategories	Value/n	CIN2+	*P*
Age, mean ± SD		42.5 ± 9.8	42.4 ± 9.8	.45^a^
Cytology	NILM	136	96 (70.6%)	.00^b^
	ASC‐US	454	341 (75.1%)	
	LSIL	319	244 (76.5%)	
	ASC‐H	67	62 (92.5%)	
	HSIL	185	171 (92.4%)	
HR‐HPV				
Group type 1^d^	Negative	72	46 (63.9%)	.00^c^
	Positive	1089	868 (79.7%)	
Group type 2^e^	Negative	72	46 (63.9%)	.00^c^
	HPV16	290	262 (90.3%)	
	HPV18	36	22 (61.1%)	
	HPV31	31	24 (77.4%)	
	HPV33	81	69 (85.2%)	
	HPV52	107	65 (60.7%)	
	HPV58	180	156 (86.7%)	
Group type 3^f^	Negative	72	46 (63.9%)	.00^c^
	Single type infection with HPV16 or 18	326	284 (87.1%)	
	Coinfection with HPV16/18	156	128 (82.1%)	
	Coinfection without HPV16/18	121	93 (76.9%)	

ASC‐H, atypical squamous cells‐cannot exclude high‐grade squamous intraepithelial lesions; ASC‐US, atypical squamous cells of undetermined significance; CIN2+, cervical intraepithelial neoplasia 2 or severer; NILM, negative for intraepithelial lesion or malignancy; HSIL, high‐grade squamous intraepithelial lesions; HPV, human papillomavirus; HR‐HPV, high‐risk HPV; LSIL, low‐grade squamous intraepithelial lesions; OR, odds ratio.

a *P* by Student's *t* test.

b *P* by Kruskal‐Wallis test.

c *P* by Chi‐square test.

d Group type 1 included 2 groups: HR‐HPV–positive and HR‐HPV–negative.

e Group type 2 included 7 groups: HR‐HPV–negative and single type infection with HPV16, 18, 31, 33, 52 or 58.

f Group type 3 included 4 groups: HR‐HPV–negative and single type infection with HPV16 or 18, coinfection with HPV16/18 and coinfection without HPV16/18.

In multivariate logistic regression models, using CIN2+ compared with CIN1 as the multivariate dependent variable, there were 3 models according to the group types of HR‐HPV (Table 4). As presented in model 1, CIN2+ had greater relationship with ASC‐H (OR = 5.20, 95% CI = 1.94‐13.90), HSIL (OR = 5.16, 95% CI = 2.67‐9.97) and positive HR‐HPV (OR = 2.10, 95% CI = 1.25‐3.51). In model 2, CIN2+ was significantly associated with single type infection with HPV16 (OR = 5.10, 95% CI = 2.68‐9.70), 33 (OR = 3.09, 95% CI = 1.39‐6.84), and 58 (OR = 3.58, 95%CI = 1.85‐6.89) after adjustment for cytology. And in model 3, CIN2+ was significantly associated with single type infection with HPV16 or 18 (OR = 3.44, 95%CI = 1.87‐6.32) and coinfection with HPV16/18 (OR = 2.58, 95% CI = 1.35‐4.92) by cytology adjusted. There was no significant difference between coinfection without HPV16/18.

**Table 4:** Multivariate logistic regression analysis of risk factors for CIN2+

Categories	Subcategories	*P*	OR	95% CI	
				Lower limit	Upper limit
Model 1 (n = 1161)^a^					
Cytology	NILM	Ref			
	ASC‐US	.19	1.33	0.87	2.04
	LSIL	.16	1.38	0.88	2.17
	ASC‐H	.00	5.20	1.94	13.90
	HSIL	.00	5.16	2.67	9.97
HR‐HPV	Negative	Ref			
	Positive	.00	2.09	1.25	3.51
Model 2 (n = 797)^b^					
HR‐HPV	Negative	Ref			
	HPV16	.00	5.10	2.68	9.70
	HPV18	.96	0.98	0.42	2.30
	HPV31	.19	1.95	0.72	5.27
	HPV33	.01	3.09	1.39	6.84
	HPV52	.80	0.92	0.49	1.74
	HPV58	.00	3.57	1.85	6.89
Model 3 (n = 675)^c^					
HR‐HPV	Negative	Ref			
	Single type infection with HPV16 or 18	.00	3.44	1.87	6.32
	Coinfection with HPV16/18	.00	2.58	1.35	4.92
	Coinfection without HPV16/18	.08	1.79	0.93	3.44

AGC, atypical glandular cells; ASC‐H, atypical squamous cells‐cannot exclude high‐grade squamous intraepithelial lesions; ASC‐US, atypical squamous cells of undetermined significance; CIN2+, cervical intraepithelial neoplasia 2 or severer; CIN1, cervical intraepithelial neoplasia 1; LSIL, low‐grade squamous intraepithelial lesions; HSIL, high‐grade squamous intraepithelial lesions; HPV, human papillomavirus; HR‐HPV, high‐risk HPV; NILM, negative for intraepithelial lesion or malignancy; OR, odds ratio.

a Model 1 included 2 HR‐HPV groups: HR‐HPV–positive and HR‐HPV–negative patients.

b Model 2 included 7 HR‐HPV groups: HR‐HPV–negative and single type infection with HPV16, 18, 31, 33, 52 or 58.

c Model 3 included 4 HR‐HPV groups: HR‐HPV–negative and single type infection with HPV16 or 18, coinfection with HPV16/18 and coinfection without HPV16/18.

## 4 DISCUSSION

As etiologic cause of cervical cancer, HPV has been studied in many ways including its prevalence and genotype distribution. The results of relative studies were varied because of different regions, races, age groups, and different methodologies or assays used.^23^, ^25^ This study mainly adds to the growing literature on the prevalence and distribution of HR‐HPV genotypes in CIN. In 1166 women with CIN, HPV16 was the most common HR‐HPV genotype which was also found in CIN2+ group. Except the reason that HPV16 is 1 indicator of colposcopic referral, a large number of studies have proven its high prevalence rate in cervical cancer and cervical precursor lesions.^5^, ^26^, ^27^ Followed by HPV16, the prevalence of HPV58, 52, 33, and 31 was ranked top 5. HPV18 was relatively rare than this 5 genotypes. And the high prevalence of HPV58 and 52 was also conformed in other researches from the same province regardless of general women or patients with CIN or invasive cervical carcinoma.^25^, ^28^, ^29^ HPV33 and 31 were also common in some regions of China.^30^ So including HPV31, 33, 52, and 58 in vaccine targeting virus genotypes might protect women from most of HPV infection in Chinese mainland. The introduction of 9vHPV vaccine may work in the future.

In concordance with previous researches, our data reported that HR‐HPV prevalence rate was increased with the severity of CIN.^31^, ^32^ And it showed that HPV16, 33 and 58 increased the risk for CIN2+ compared with HR‐HPV–negative patients. There was a population‐based study supported that the prevalence of persistent HPV infection was also increased with the severity of cervical lesion and showed HPV16, 58, 18, 52, and 33 were most common in persistent infection.^31^ And a population‐based, prospective observational study suggested that HPV16/18/31/33/52/58 infection could be referred to CDB immediately.^33^ In addition, Ying et al reported that HPV16 and 58 were closely associated with CIN2/3 and squamous cervical cancer compared with cervicitis.^21^ So in HR‐HPV‐positive women, we assume that it is necessary to pay more attention to the women who infected by HPV33 and 58. And it is needed to be studied further that whether the oncogenicity of 33 and 58 is similar as HPV16.

Up to now, many researchers had studied the association between CIN2+ and infection with multiple HPV types.^34^, ^35^ Several previous studies had reported that infection with multiple HR‐HPV genotypes increased the risk of development and/or progression of CIN and cervical cancer.^36^, ^37^ And to answer whether such association was due to single addictive risk or synergistic interaction between multiple HPV types, a study including 5871 women aged 18‐25 years indicated that coinfecting HPV genotypes led to CIN independently and its results showed the association of HR‐HPV coinfection with risk of CIN2+ was largely driven by HPV16.^37^ On the contrary, another study found that due to the synergistic interaction of infection with multiple HR‐HPV types, the risk of CIN increased despite HPV16.^38^ However, there were only a few studies had assessed the risk of CIN2+ caused by infection with multiple HPV types according to the occurrence of HPV16/18. In the present study, the results suggested that HPV16/18 could increase the risk for CIN2+ whatever single or multiple HR‐HPV type infection it was. And there was no evidence to support multiple HR‐HPV type infection without HPV16/18 could increase the risk for CIN2+. And it was interested that single type infection with HPV16 or 18 had relatively higher risk than multiple type infection. Whether interaction among different genotypes decrease or increase the carcinogenesis of HPV16/18 needs to be estimated in more detail and in large population.

This study provides reference data mainly for HR‐HPV genotype distribution in CIN and the association between HR‐HPV and CIN2+. HR‐HPV–positive women remain at an elevated risk for CIN2+. According to the high prevalence of top 6 HR‐HPV genotypes (HPV16, 18, 31, 33, 52, and 58), we recommend these HR‐HPV genotypes have the priority to be included in second‐generation prophylactic vaccines. And women who infected by HPV33 or 58 may have higher risk for CIN2+ and need be heeded. The role of infection with multiple HR‐HPV types showed in the study triggers an intensive study by differentiating patients according to the presence of HPV16/18. And the question whether there is interaction among HR‐HPV genotypes in infection with multiple HPV types needs to be studied further.

As a retrospective study, there are several limitations. The patients of this study were mainly from Wenzhou city which could not represent all women in China. And it is necessary to carry multicenter cooperation to study it further.

## CONFLICT OF INTEREST

The authors declare that they have no conflict of interest.
